# The Role of Surgical Resection Following Tyrosine Kinase Inhibitors Treatment in Patients with Advanced Gastrointestinal Stromal Tumors: A Systematic Review and Meta-analysis

**DOI:** 10.7150/jca.30040

**Published:** 2019-10-06

**Authors:** Yinghao Guo, Jinqiang Liu, Fei Wang, Qiao Wang, Gaozan Zheng, Shushang Liu, Xiao Lian, Hongwei Zhang, Fan Feng

**Affiliations:** 1 Department of Digestive Surgery, Xijing Hospital of Digestive Diseases, the Fourth Military Medical University, 127 West Changle Road, 710032, Xi'an, Shaanxi, China.; 2 Health company, 92667 Army of PLA, 39 East Zaoshan Road, 266100, Qingdao, Shandong, China.; 3 Cadre' s sanitarium, 62101 Army of PLA, 67 Nahu Road, 464000, Xinyang, Henan, China.; 4 Department of General Surgery, No. 534 Hospital of PLA, West Lichun Road, 471000, Luoyang, Henan, China.; 5 Department of General Surgery, No. 91 Hospital of PLA, 239 Gongye Road, 454000, Jiaozuo, Henan, China.

**Keywords:** Gastrointestinal stromal tumor, Tyrosine kinase inhibitor, Surgery, Meta-analysis

## Abstract

**Background** The benefit of surgical resection for advanced gastrointestinal stromal tumors (GISTs) following tyrosine kinase inhibitors (TKIs) treatment was still under debate. The present meta-analysis was designed to assess the value of surgical resection for the prognosis of patients with metastatic, recurrence and unresectable GISTs. **Methods** A systematic search of PubMed Central, PubMed, EMBASE and the Cochrane Library database was performed. Relevant studies of the role of surgery in advanced GISTs published before 1 May 2019 were identified. The quality of studies was assessed by the Newcastle-Ottawa Quality Assessment Scale. The progression-free survival (PFS) and overall survival (OS) were assessed through software Stata 15.0. **Results** A total of 6 retrospective studies including 655 patients were analyzed. The pooled result revealed that surgical resection group was associated with better PFS (HR = 2.08; 95% CI: 1.58 to 2.76; P<0.001) and better OS (HR = 2.13; 95% CI: 1.59 to 2.85; P<0.001) compared with TKIs treatment alone group. **Conclusions** Surgical resection following TKIs treatment could significantly improve the prognosis of patients with advanced GISTs.

## Introduction

GISTs are the commonest mesenchymal tumors of the gastrointestinal tract, with an incidence range from 6.8 to 19.7 per million 1-3. GISTs can arise anywhere within the gastrointestinal tract but most commonly in the stomach, followed by small intestine 4, 5. Approximately 85% of GISTs harbor a gain-of-function mutation in either KIT or platelet-derived growth factor receptor alpha (PDGFRA) genes 6-8. GISTs could display a broad spectrum of clinical behavior, from benign to malignant 9.

For localized GISTs, complete resection remains the standard treatment, and surgery combination with TKIs treatment could significantly improve survival of patients. However, approximately 30% to 50% of patients will suffer from recurrence or metastasis within 3 years in absence of TKIs therapy 10-12. Moreover, up to 50% of GISTs are metastatic or unresectable at the time of diagnosis^4^, ^13^. For these patients, imatinib was considered as first line treatment. The induction of imatinib has significantly improved the survival of patients with metastatic, recurrent and unresectable GISTs^14^-^16^. However, approximately 15% of patients were primarily resistant to imatinib treatment^17^, ^18^. Although up to 80% of patients were initially responsive to imatinib treatment, most patients will develop secondary resistance resulting from secondary mutations within 2 years^19^, ^20^. In addition, approximately 20% of patients do not tolerate TKIs treatment^21^-^23^. Thus, surgical resection may be an additional option for metastatic, recurrent or unresectable GISTs, as it could decrease the tumor burden of patients. Over the last two decades, a series of studies^24^-^28^ have demonstrated that surgical resection could improve the clinical outcomes of patients with advanced GISTs following TKIs therapy. Nevertheless, conflicting data has also been reported^29^.

Therefore, the present study aims to investigate the clinical benefits of surgical resection following TKIs treatment for patients with metastatic, recurrent or unresectable GISTs through a systematic review and meta-analysis.

## Methods

A systematic electronic search of PubMed Central, PubMed, EMBASE and the Cochrane Library was performed with language restriction to English to identify eligible studies published from the inception dates to May 1, 2019, utilizing the following combined Medical Subject Headings (MeSH) terms and relevant text words: 'gastrointestinal stromal tumors', 'GIST', 'GISTs', 'recurrence', 'advanced', 'metastasis', 'surgery', 'resection', 'cytoreduction,' and 'palliative'. Additionally, reference lists of review articles, commentaries, editorials, identified studies and conference proceedings were hand searched and cross-referenced to identify any other relevant data.

Two reviewers (GYH and LJQ) independently assessed the studies using the following inclusion criteria: (1) Diagnosed as recurrent, metastatic or unresectable GISTs; (2) The studies contain two groups: surgical resection group (S group) and TKIs treatment alone group (NS group); (3) In S group, surgical resection should be performed following TKIs treatment; (4) The informative data were available; and (5) Published in English. Disagreements were settled by consultation or adjudicated by a third reviewer (WF). Titles and abstracts were used to screen for initial study inclusion. Full-text review was performed when abstracts were insufficient to determine if the study met inclusion criteria or not.

Two researchers (ZGZ and LSS) independently extracted the following variables from each included study: the first author, years of survey, country of origin, study design, sample size, follow-up duration, outcomes (OS and PFS), median age, gender, primary tumors sites, metastases site, response to TKIs and genotype.

The quality of the included papers was assessed using the Newcastle-Ottawa Quality Assessment Scale (NOS) 30. The risk of bias was deemed to be high if a study scored 0-3, moderate if it scored 4-6 and low if it scored 7-9.

## Statistical analysis

This meta-analysis was performed according to the Preferred Reporting Items for Systematic Reviews and Meta-Analyses (PRISMA) guidelines^31^ (Supplementary Table S1). The primary outcomes were progression-free survival (PFS) and overall survival (OS). Hazard ratio (HR) and 95% confidence intervals (CIs) were used to analysis time-to-event outcomes, if not reported, we derived HR and 95% CIs based on data reconstructed from Kaplan-Meier survival curves reported by Tierney et al^32^. The surgical resection group was settled as “reference” group. A HR >1 indicated a worse prognosis in patients with advanced GISTs. Heterogeneity were estimated using the I^2^ statistic and the Cochrane Q test^33^. An I^2^ statistic was interpreted to reflect low heterogeneity (0%-25%), moderate heterogeneity (26%-75%), and high heterogeneity (76%-100%), as was a P value of less than or equal to 0.05 for heterogeneity. Where there was a moderate or high likelihood of heterogeneity, sensitivity analyses were done to seek the reasons for the differences. The fixed-effect model was first fitted for all outcomes, if the p value of the heterogeneity Q test was greater than 0.1 (I^2^≤40), the random effects model was used.

Publication bias was assessed by visual inspection of the funnel plot. The Begg's^34^ and Egger's test^35^ were used to identify asymmetry of funnel plots and significant publication bias was defined as a p value <0.1.

Statistical software Stata (version 15.0) was used for data management and analysis. A two-sided p value less than 0.05 were considered statistically significant.

## Results

### Enrolled studies and quality assessment

**Figure 1:**
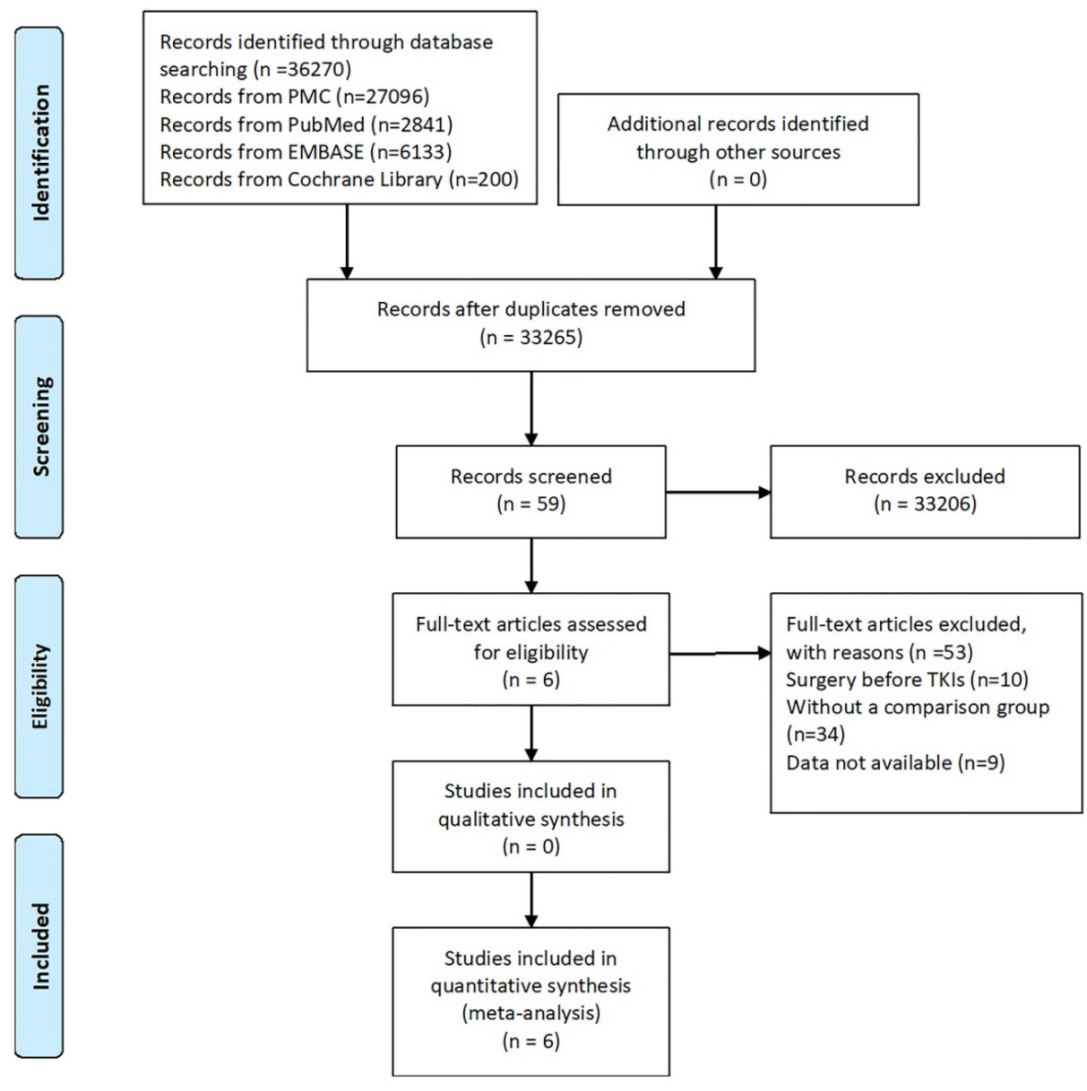
Flow chart of literature selection process.

A total of 36270 studies (PubMed Central, n=27096; PubMed, n=2841; EMBASE, n=6133; Cochrane library, n=200) were identified using our search strategy, of which six retrospective studies^36^-^41^ were included in this meta-analysis. A flow chart of the search strategy and reasons for exclusion are illustrated in Figure 1.

The 6 studies enrolled 655 patients, of which 239 patients underwent surgery following TKIs therapy and 416 patients underwent TKIs treatment alone. The publication year ranged from 2005 to 2018. The recruitment time was between 2001 and 2016. Characteristics of the included studies were provided in Table 1 (Supplementary Table S2).

The quality assessment results of the included studies are shown in Table 2. All six articles were scored≥7, which ensured the high quality of the included articles.

### Survival outcomes

**Figure 2:**
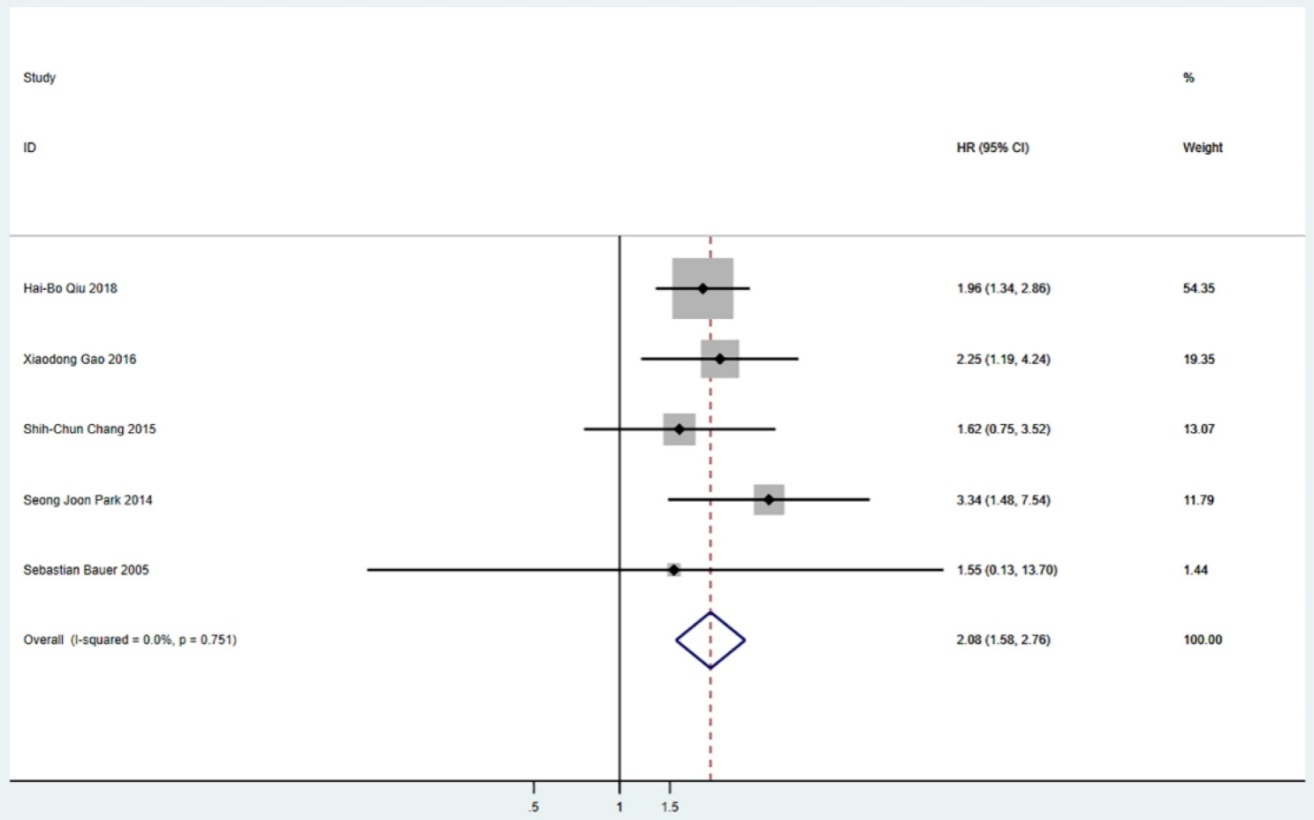
Meta-analysis of PFS between S group and NS group. PFS=progression-free survival, HR=hazard ratio, CI=confidence interval, I-squared=the percentage of total variation across studies that is due to heterogeneity rather than chance.

**Figure 3:**
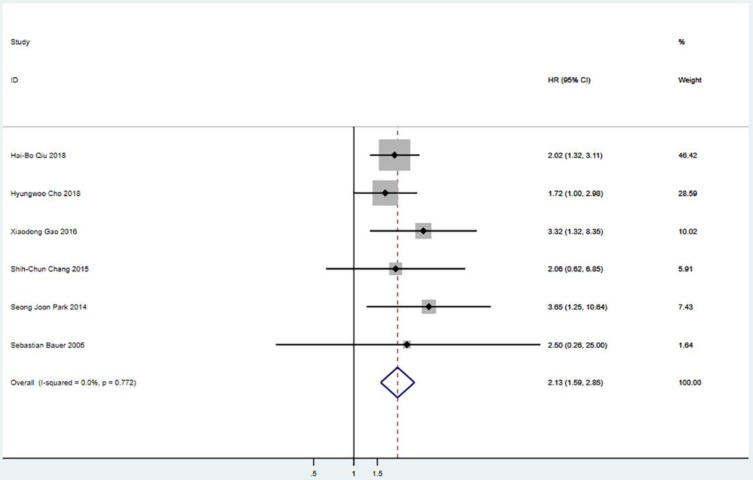
Meta-analysis of OS between S group and NS group. OS=overall survival, HR=hazard ratio, CI=confidence interval, I-squared=the percentage of total variation across studies that is due to heterogeneity rather than chance.

Five studies including 565 patients reported PFS. The pooled analysis revealed a better PFS for the surgical resection group than that in the TKIs treatment alone group (HR = 2.08; 95% CI: 1.58 to 2.76; P<0.001) (Figure 2). Six studies including 655 patients suggested that surgical resection group was associated with a better OS compared to TKIs treatment alone group (HR = 2.13; 95% CI: 1.59 to 2.85; P<0.001) (Figure 3).

### Publication bias

**Figure 4:**
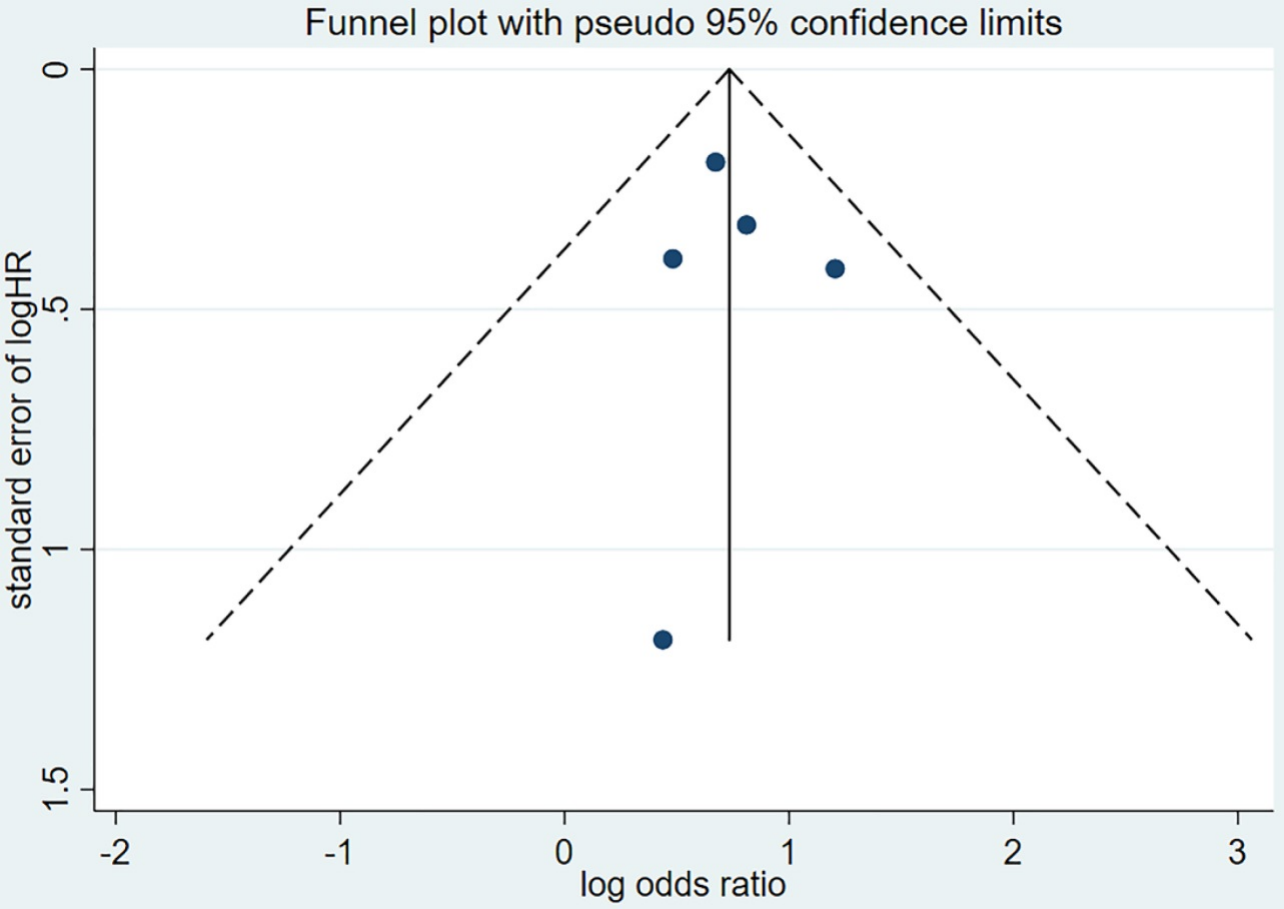
Funnel plot of hazard ratio for PFS. PFS=progression-free survival, HR=hazard ratio.

**Figure 5:**
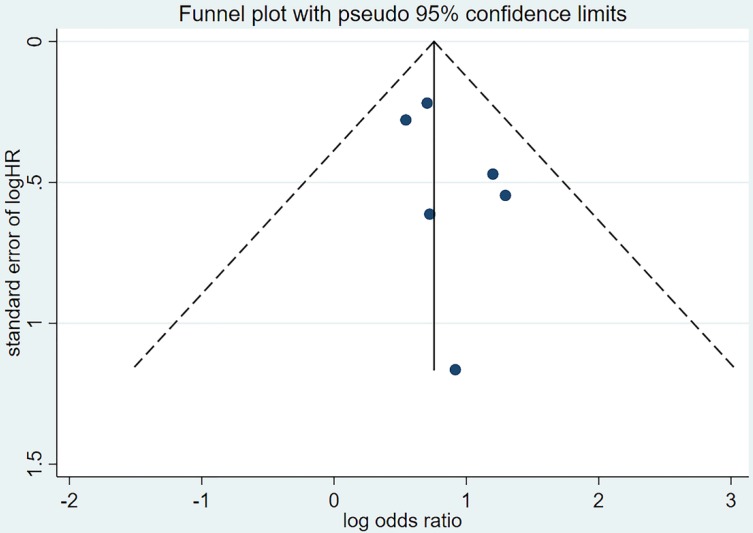
Funnel plot of hazard ratio for OS. OS=overall survival, HR=hazard ratio.

Publication bias was evaluated based on the funnel plot (Figure 4,5) using the Begg's and Egger's test. No publication bias was identified in the six studies.

## Discussion

Despite most GISTs patients initially benefit from TKIs therapy, secondary resistance occurs at a median time of 2 years, which result in poor prognosis. Therefore, surgical resection for advanced GISTs is thought to be an additional therapy to remove residual tumor or drug resistant clones to induce remission or curation. However, the value of surgical resection for this situation was still under debate. Thus, the present meta-analysis was performed to evaluate the value of surgical resection for the prognosis of patients with metastatic, recurrent, or unresectable GISTs. Our pooled analysis demonstrated that GIST patients who treated with surgery following TKIs therapy showed better clinical outcomes in terms of PFS and OS compared with that received TKIs therapy alone.

Although randomized controlled trials (RCTs) are the first choice for meta-analysis, the RCTs focused on this point are insufficient for meta-analysis. One from the European Organization for Research and Treatment of Cancer (EORTC, NCT00956072) [42]aimed to evaluate the value of cytoreductive surgery for imatinib-sensitive GIST patients was stopped after recruiting only 12 patients without any conclusions. One from China (CTR-TRC-000000344) 43 compared the survival of patients with peritoneal metastasis between surgery group (19 patients) and imatinib treatment alone group (22 patients) also terminated due to poor accrual. However, the PFS showed a trend towards survival benefit in the surgery arm, but no statistically significant difference was reached. The other one from China 44 compared the survival of patients with liver metastasis between surgery group (19 patients) and imatinib treatment alone group (20 patients) suggested that surgery combined with imatinib treatment could significantly improve the OS of patients, especially in poor responders. The results of the two RCTs were consistent with our present study.

The timing of surgery was also very important. The European Society for Medical Oncology (ESMO) practical guidelines^45^ suggested that surgery should be performed at the time of maximal tumor response, generally 6-12 months after TKIs treatment for localized GISTs. However, optimal timing of surgery for advanced GISTs was still hard to ascertain. Keung et al.^46^ recommended that cytoreductive surgery of residual metastatic disease should be considered no earlier than 6 months and no later than 2 years after TKIs initiation. Bischof et al. [47]suggested that patients with partial response (PR) and stable disease (SD) treated with TKIs therapy and surgery showed significantly prolonged PFS and OS compared with those with progressive disease (PD). Qiu et al.^36^ also reported the similar results. These findings indicated that PR and SD may be the proper time for cytoreductive surgery. Although the prognosis of patients with PD receiving cytoreductive surgery was not satisfactory, no study has compared the survival of patients with PD between surgical treatment and TKIs treatment alone. Thus, selection of optimal candidates for cytoreductive surgery is also very difficult in clinical practice.

Up to date, no study has definitely evaluated the value of R2 cytoreductive surgery following TKIs treatment for advanced GISTs patients. All the enrolled six studies did not show the benefit of R2 resection through subgroup analysis due to extremely small sample size. Thus, the value of R2 resection was unclear, and the prolonged survival of patients with surgery (R0/R1/R2) may result from improved survival of patients receiving R0/R1 resection. Moreover, even if R2 resection could improve the prognosis of patients, enough attention should be paid to the degree of cytoreduction, because low cytoreduction proportion may not benefit the survival of patients with advanced GISTs. Unfortunately, the degree of cytoreduction was not described in the six studies included in our present study.

It is well known that type of gene mutation was associated with the prognosis of GIST patients^48^, ^49^. However, the impact of gene mutational status on the clinical outcomes of cytoreductive surgery is still unknown. Qiu et al.^36^ reported that patients with primary c-KIT exon 11 rather than exon 9 mutation could benefit from cytoreduction surgery. Result of Park et al. 39 revealed that c-Kit exon 11 rather than exon 9 or wild-type was associated with longer PFS in the surgery group.

Continuation of TKIs therapy is very critical for advanced GIST patients after cytoreductive surgery. Both the National Comprehensive Cancer Network (NCCN) guidelines^50^ and ESMO practical guidelines^45^ recommend continuing imatinib mesylate (IM) as a post-resection treatment for advanced GIST patients. Zhang et al. 51 compared the clinical outcomes of advanced GIST patients receiving imatinib or switching to sunitinib after cytoreductive surgery based on 97 patients from 13 centers. They found that switching to sunitinib could significantly improve the PFS of patients compared with continuing imatinib treatment after R0 or cytoreductive surgery (30 months vs 12 months, p=0.009), which indicated that sunitinib may be superior to imatinib for the patients after cytoreductive surgery. However, this should be confirmed through further studies based on larger populations.

So far, there was only one meta-analysis evaluated the role of surgery in patients with advanced GIST, which included 9 studies with 1416 patients. The pooled results revealed that surgery combined with TKIs therapy is associated with a better OS and PFS. However, patients in 4 studies included in this meta-analysis received surgery prior to imatinib therapy, which may result in bias and finally influence the evaluation of value of surgery in advanced GISTs patients. Because cytoreductive surgery before imatinib treatment for advanced GISTs patients was not recommended in the NCCN and ESMO guidelines^45^, ^50^. Therefore, our present meta-analysis strictly limited the inclusion criteria and updated the relevant literatures.

There are several limitations in our study. First, the sample size was relatively small; only six retrospective studies were analyzed. Second, the essential selection bias of the non-randomized and retrospective studies may result in incomparability between the two groups. For instance, the baseline characteristics in some studies were not homogeneous especially in physical condition, metastatic site, tumor size and number of metastases. Third, subgroup analysis concerning R2 resection, timing of surgery, and type of gene mutation, etc. was not performed due to data insufficiency in the included studies. To overcome these limitations, prospective randomized controlled trials based on large populations should be conducted.

## Conclusion

In conclusion, this meta-analysis has demonstrated that surgical resection following TKIs therapy could prolong survival of patients with metastatic, recurrent or unresectable GISTs when compared with TKIs therapy alone. Cytoreductive surgery could be considered for selected advanced GIST patients after TKIs therapy.

## Supplementary Material

Supplementary figures and tables.

## Figures and Tables

**Table 1:** Summary of studies included in the meta-analysis

Author	Year	Study period	Country	Study design	Sample size			Follow-ups	Outcomes
					Total	S group	NS group	Median, range	
Hai-Bo Qiu	2018	2002-2008	China	Retro	156	87	69	23.7, 3-81.5	PFS OS
Hyungwoo Cho	2018	2003-2016	Korea	Retro	90	38	52	31.0, NR	TTF OS
Xiaodong Gao	2016	2005-2014	China	Retro	57	38	19	26.0, 8-104	PFS OS
Shih-Chun Chang	2015	2001-2013	Taiwan	Retro	128	22	106	NR	PFS OS
Seong Joon Park	2014	2001-2010	Korea	Retro	134	42	92	58.9, 15.4-129.1	PFS OS
Sebastian Bauer	2005	2001-2004	Germany	Retro	90	12	78	29.8, 17-41	PFS OS

Retro: retrospective study; TTF: time to IM treatment failure; PFS: progression-free survival; OS: overall survival; NR: not reported.

**Table 2:** Newcastle-Ottawa Scale Assessment of enrolled studies

Ref	Selection (0-4)				Comparability (0-2)		Outcome (0-3)			Total
	REC	Snec	AE	OINP	SCB	SCA	AO	FU	AFC	
Hai-Bo Qiu	1	1	1	1	1	1	1	1	1	9
Hyungwoo Cho	1	1	1	1	0	0	1	1	1	7
Xiaodong Gao	1	1	1	1	1	0	1	1	1	8
Shih-Chun Chang	1	1	1	1	0	1	1	1	1	8
Seong Joon Park	1	1	1	1	0	0	1	1	1	7
Sebastian Bauer	1	1	1	1	1	0	1	1	1	8

REC: Representativeness of the exposed cohort; SNEC: Selection of the no exposed cohort; AE: Ascertainment of exposure; OINP: Outcome of interest not presented in the start of study; SCB: Study controls for basic characteristics; SCA: Study controls for additional factor; AO: Assessment of outcome; FU: Follow-up; AFC: Adequacy of follow up.

## Abbreviations

GIST: gastrointestinal stromal tumor
TKIs: tyrosine kinase inhibitors
PFS: progression-free survival
OS: overall survival
HR: hazard ratio
CI: confidence interval
PDGFRA: platelet-derived growth factor receptor alpha
MeSH: Medical Subject Headings
S group: surgical resection group
NS group: TKIs treatment alone group
PR: partial response
SD: stable disease
PD: progressive disease
NOS: Newcastle-Ottawa Quality Assessment Scale
ASCO: American Society of Clinical Oncology
EORTC: European Organization for Research and Treatment of Cancer
ESMO: European Society for Medical Oncology
NCCN: National Comprehensive Cancer Network
