# Supplementary material for: The Role of Surgical Resection Following Tyrosine Kinase Inhibitors Treatment in Patients with Advanced Gastrointestinal Stromal Tumors: A Systematic Review and Meta-analysis
Source: J Cancer. 2019 Oct 6;10(23):5785–92. doi: 10.7150/jca.30040 (PMC6843877; doi:10.7150/jca.30040)
Supplement: Supplementary file 1 — Supplementary figures and tables. [file jcav10p5785s1.pdf]

Supplementary File: search strategy in detail.

PMC：N=27096

(((((((((((((((((("Gastrointestinal Stromal Tumors"[Mesh]) OR gastrointestinal stromal tumors) OR stromal tumor, gastrointestinal) OR stromal tumors, gastrointestinal) OR tumor, gastrointestinal stromal) OR tumors, gastrointestinal stromal) OR gastrointestinal stromal neoplasms) OR neoplasm, gastrointestinal stromal) OR neoplasms, gastrointestinal stromal) OR stromal neoplasm, gastrointestinal) OR stromal neoplasms, gastrointestinal) OR gastrointestinal stromal tumor) OR gastrointestinal stromal neoplasm) OR gastrointestinal stromal sarcoma) OR gist) OR gists)) AND ((((((((((((((((((((((("Recurrence"[Mesh]) OR recurrence) OR recurrences) OR recrudescence) OR relapse) OR relapses) OR recur) OR recidivate) OR recidivating) OR recrudescences) OR recurrent) OR advanced) OR terminal) OR late stage) OR unresectable) OR metastasis) OR metastases) OR metastatic) OR refractory) OR obstinacy) OR intractable) OR resistance) OR resistant)) AND ((((((((((((((((((((((((((((((((((((("surgery"[Subheading]) OR operative therapy) OR operative procedures) OR invasive procedures) OR operations) OR preoperative procedures) OR perioperative procedures) OR preoperative procedures) OR intraoperative procedures) OR resection) OR surgery) OR surgical) OR excision) OR gastrectomy) OR gastrectomies) OR "Cytoreduction Surgical Procedures"[Mesh]) OR cytoreduction surgical procedures) OR cytoreduction surgical procedure) OR procedure, cytoreduction surgical) OR surgical procedure, cytoreduction) OR debulking surgical procedures) OR debulking surgical procedure) OR procedure, debulking surgical) OR surgical procedure, debulking) OR cytoreductive surgery) OR cytoreductive surgeries) OR surgery, cytoreductive) OR cytoreductive surgical procedures) OR cytoreductive surgical procedure) OR procedure, cytoreductive surgical) OR surgical procedure, cytoreductive) OR surgical procedures, cytoreductive) OR cytoreductive) OR cytoreduction) OR reduction) OR reductive) OR palliative)

PubMed：N=2841

((((((((((((((((("Gastrointestinal Stromal Tumors"[Mesh]) OR gastrointestinal stromal tumors) OR stromal tumor, gastrointestinal) OR stromal tumors, gastrointestinal) OR tumor, gastrointestinal stromal) OR tumors, gastrointestinal stromal) OR gastrointestinal stromal neoplasms) OR neoplasm, gastrointestinal stromal) OR neoplasms, gastrointestinal stromal) OR stromal neoplasm, gastrointestinal) OR stromal neoplasms, gastrointestinal) OR gastrointestinal stromal tumor) OR gastrointestinal stromal neoplasm) OR gastrointestinal stromal sarcoma) OR gist) OR gists)) AND ((((((((((((((((((((((("Recurrence"[Mesh]) OR recurrence) OR recurrences) OR recrudescence) OR relapse) OR relapses) OR recur) OR recidivate) OR recidivating) OR recrudescences) OR recurrent) OR advanced) OR terminal) OR late stage) OR unresectable) OR metastasis) OR metastases) OR metastatic) OR refractory) OR obstinacy) OR intractable) OR resistance) OR resistant)) AND ((((((((((((((((((((((((((((((((((((("surgery"[Subheading]) OR operative therapy) OR operative procedures) OR invasive procedures) OR operations) OR preoperative procedures) OR perioperative procedures) OR preoperative procedures) OR intraoperative procedures) OR resection) OR surgery) OR surgical) OR excision) OR gastrectomy) OR gastrectomies) OR "Cytoreduction Surgical Procedures"[Mesh]) OR cytoreduction surgical procedures) OR cytoreduction surgical procedure) OR procedure, cytoreduction surgical) OR surgical procedure, cytoreduction) OR debulking surgical procedures) OR debulking surgical procedure) OR procedure, debulking surgical) OR surgical procedure, debulking) OR cytoreductive surgery) OR cytoreductive surgeries) OR surgery, cytoreductive) OR cytoreductive surgical procedures) OR cytoreductive surgical procedure) OR procedure, cytoreductive surgical) OR surgical procedure, cytoreductive) OR surgical procedures, cytoreductive) OR cytoreductive) OR cytoreduction) OR reduction) OR reductive) OR palliative)

EMBASE: N=6133

('gastrointestinal stromal tumor'/exp OR 'gastrointestinal stromal tumors' OR 'stromal tumor, gastrointestinal' OR 'stromal tumors, gastrointestinal' OR 'tumor, gastrointestinal stromal' OR 'tumors, gastrointestinal stromal' OR 'gastrointestinal stromal neoplasms' OR 'neoplasm, gastrointestinal stromal' OR 'neoplasms, gastrointestinal stromal' OR 'stromal neoplasm, gastrointestinal' OR 'stromal neoplasms, gastrointestinal' OR 'gastrointestinal stromal tumor' OR 'gastrointestinal stromal neoplasm' OR 'gastrointestinal stromal sarcoma' OR 'gist' OR 'gists') AND ('recurrent disease'/exp OR 'recurrent disease' OR 'recurrence' OR 'recurrences' OR 'recrudescence' OR 'recrudescences' OR 'relapse' OR 'relapses' OR 'recur' OR 'recidivate' OR 'recidivation' OR 'recurrent' OR 'advanced' OR 'terminal' OR 'late stage' OR 'unresectable' OR 'metastasis' OR 'metastases' OR 'metastatic' OR 'refractory' OR 'obstinacy' OR 'intractable' OR 'resistance' OR 'resistant') AND ('surgery'/exp OR 'surgery' OR 'operative therapy' OR 'operative procedures' OR 'invasive procedures' OR 'operations' OR 'peroperative procedures' OR 'perioperative procedures' OR 'preoperative procedures' OR 'intraoperative procedures' OR 'resection' OR 'surgical' OR 'excision' OR 'gastrectomy' OR 'gastrectomies' OR 'cytoreduction'/exp OR 'cytoreduction surgical procedure' OR 'procedure, cytoreduction surgical' OR 'surgical procedure, cytoreduction' OR 'debulking surgical procedures' OR 'debulking surgical procedure' OR 'procedure, debulking surgical' OR 'surgical procedure, debulking' OR 'cytoreductive surgery' OR 'cytoreductive surgeries' OR 'surgery, cytoreductive' OR 'cytoreductive surgical procedures' OR 'cytoreductive surgical procedure' OR 'procedure, cytoreductive surgical' OR 'surgical procedure, cytoreductive' OR 'surgical procedures, cytoreductive' OR 'cytoreductive' OR 'cytoreduction' OR 'reduction' OR 'reductive' OR 'palliative') AND [english]/lim

Cochrane：N=200

ID	Search	Hits
#1	MeSH descriptor: [Gastrointestinal Stromal Tumors] explode all trees	128
#2	(gastrointestinal) (Word variations have been searched)	45728
#3	(stromal) (Word variations have been searched)	2066
#4	(Tumor or Neoplasm or Sarcoma) (Word variations have been searched)	118386
#5	#2 and #3 and #4	443
#6	GIST or GISTS	506
#7	#1 or #5 or #6	688
#8	MeSH descriptor: [Recurrence] explode all trees	11477
#9	Recurrence or Recurrences or Recrudescence or Recrudescences or Relapse	64288
#10	Relapses or Recur or Recidivate or Recidivation or recurrent	36607
#11	advanced or terminal or late stage or unresectable or Neoplasm Metastasis	71525
#12	MeSH descriptor: [Neoplasm Metastasis] explode all trees	4728
#13	metastasis or metastases or metastatic or refractory or obstinacy	53497
#14	intractable or resistance or resistant	69159
#15	#8 or #9 or #10 or #11 or #12 or #13 or #14	228514
#16	MeSH descriptor: [General Surgery] explode all trees	334
#17	Surgery or General Surgery or operative therapy or operative procedures or invasive procedures	224247
#18	operations or peroperative procedures or perioperative procedures or preoperative procedures or intraoperative procedures	18710
#19	resection or surgical or excision or gastrectomy or gastrectomies	111387
#20	Cytoreduction or Procedure, Cytoreduction Surgicalor Debulking Surgical Procedures or Debulking Surgical Procedure or Procedure, Debulking Surgical	434
#21	Surgical Procedure, Debulking or Cytoreductive Surgery or Cytoreductive Surgeries or Surgery, Cytoreductive or Cytoreductive Surgical Procedures	745
#22	Cytoreductive Surgical Procedure or Procedure, Cytoreductive Surgical or Surgical Procedure, Cytoreductive or Surgical Procedures, Cytoreductive or cytoreductive	850
#23	cytoreduction or reduction or reductive or palliative	161562
#24	#16 or #17 or #18 or #19 or #20 or #21 or #22 or #23	384842
#25	#7 and #15 and #24	200

Supplementary Table S1: PRIAMA checklist of the study.

Section/topic	#	Checklist item	Reported on page #
**TITLE**			
Title	1	Identify the report as a systematic review, meta-analysis, or both.	page 1
**ABSTRACT**			
Structured summary	2	Provide a structured summary including, as applicable: background; objectives; data sources; study eligibility criteria, participants, and interventions; study appraisal and synthesis methods; results; limitations; conclusions and implications of key findings; systematic review registration number.	page 2
**INTRODUCTION**			
Rationale	3	Describe the rationale for the review in the context of what is already known.	page 2
Objectives	4	Provide an explicit statement of questions being addressed with reference to participants, interventions, comparisons, outcomes, and study design (PICOS).	page 3
**METHODS**			
Protocol and registration	5	Indicate if a review protocol exists, if and where it can be accessed (e.g., Web address), and, if available, provide registration information including registration number.	page 4
Eligibility criteria	6	Specify study characteristics (e.g., PICOS, length of follow-up) and report characteristics (e.g., years considered, language, publication status) used as criteria for eligibility, giving rationale.	page 3
Information sources	7	Describe all information sources (e.g., databases with dates of coverage, contact with study authors to identify additional studies) in the search and date last searched.	page 3
Search	8	Present full electronic search strategy for at least one database, including any limits used, such that it could be repeated.	page 3
Study selection	9	State the process for selecting studies (i.e., screening, eligibility, included in systematic review, and, if applicable, included in the meta-analysis).	page 3
Data collection process	10	Describe method of data extraction from reports (e.g., piloted forms, independently, in duplicate) and any processes for obtaining and confirming data from investigators.	page 4
Data items	11	List and define all variables for which data were sought (e.g., PICOS, funding sources) and any assumptions and simplifications made.	page 4
Risk of bias in individual studies	12	Describe methods used for assessing risk of bias of individual studies (including specification of whether this was done at the study or outcome level), and how this information is to be used in any data synthesis.	page 4
Summary measures	13	State the principal summary measures (e.g., risk ratio, difference in means).	page 4
Synthesis of results	14	Describe the methods of handling data and combining results of studies, if done, including measures of consistency (e.g., I^2) for each meta-analysis.	page 4

Section/topic	#	Checklist item	Reported on page #
Risk of bias across studies	15	Specify any assessment of risk of bias that may affect the cumulative evidence (e.g., publication bias, selective reporting within studies).	page 4
Additional analyses	16	Describe methods of additional analyses (e.g., sensitivity or subgroup analyses, meta-regression), if done, indicating which were pre-specified.	page 4
**RESULTS**			
Study selection	17	Give numbers of studies screened, assessed for eligibility, and included in the review, with reasons for exclusions at each stage, ideally with a flow diagram.	page 5
Study characteristics	18	For each study, present characteristics for which data were extracted (e.g., study size, PICOS, follow-up period) and provide the citations.	page 5
Risk of bias within studies	19	Present data on risk of bias of each study and, if available, any outcome level assessment (see item 12).	page 5
Results of individual studies	20	For all outcomes considered (benefits or harms), present, for each study: (a) simple summary data for each intervention group (b) effect estimates and confidence intervals, ideally with a forest plot.	page 5
Synthesis of results	21	Present results of each meta-analysis done, including confidence intervals and measures of consistency.	page 5
Risk of bias across studies	22	Present results of any assessment of risk of bias across studies (see Item 15).	page 5
Additional analysis	23	Give results of additional analyses, if done (e.g., sensitivity or subgroup analyses, meta-regression [see Item 16]).	page 5
**DISCUSSION**			
Summary of evidence	24	Summarize the main findings including the strength of evidence for each main outcome; consider their relevance to key groups (e.g., healthcare providers, users, and policy makers).	page 5
Limitations	25	Discuss limitations at study and outcome level (e.g., risk of bias), and at review-level (e.g., incomplete retrieval of identified research, reporting bias).	page 7
Conclusions	26	Provide a general interpretation of the results in the context of other evidence, and implications for future research.	page 8
**FUNDING**			
Funding	27	Describe sources of funding for the systematic review and other support (e.g., supply of data); role of funders for the systematic review.	page 8

Supplementary Table S2： Clinicalpathological features of studies included in the meta-analysis.

Clinicalpathological features	Hai-Bo Qiu		Hyungwoo Cho		Xiaodong Gao		Shih-Chun Chang		Seong Joon Park		Sebastian Bauer	
	S group	NS group	S group	NS group	S group	NS group	S group	NS group	S group	NS group	S group	NS group
**Male/Female**	63/24	43/26	NA	NA	37/7	10/9	15/7	66/40	25/17	55/37	6/6	45/33
**Age**	NR	NR	NA	NA	53	61	56.36	58.42	51	58	57	62
**Primary sites**												
Stomach	56	48	NA	NA	10	6	10	41	15	39	5	26
Small bowel	22	10	NA	NA	22	11	10	48	21	44	5	21
Others	9	11	NA	NA	6	2	2	17	6	9	2	31
**Metastases site**												
Liver	19	13	NA	NA	21	10	12	74	34	62	9	53
Peritoneum	33	22	NA	NA	24	15	9	55	12	56	6	41
Liver and Peritoneum	32	28	NA	NA								
Others	3	6	NA	NA					5	5		21
**Response to TKIs**												
CR+PR	16	15	NA	NA	NR	NR	22	65	27	68	11	39
SD	34	27	NA	NA	NR	NR	0	21	15	24	1	17
PD	37	27	NA	NA	NR	NR	0	20	0	0	0	22
**Genotype**												
c-KIT exon 11	58	44	NA	NA	27	14	NR	NR	29	60	NR	NR
c-KIT exon 9	11	8	NA	NA	5	1	NR	NR	4	10	NR	NR
Others	18	11	NA	NA	1	1	NR	NR	3	14	NR	NR
Not available			NA	NA	5	3	NR	NR	6	8	NR	NR

S group: surgical resection group; NS group: TKIs treatment alone group; CR: complete response; PR: partial response; SD: stable disease; PD: progressive disease; NR: not reported; NA: not available.
